# The Psychological Impact of the Tertiary Hospital Reappraisal on Resident Doctors in the Post-pandemic Era: A Cross-sectional Study in Ningbo

**DOI:** 10.3389/fpsyt.2021.770851

**Published:** 2022-02-11

**Authors:** Zhonghao Shao, Angyang Cao, Wenjun Luo, Yanling Zhou, Jianhua Wang, Yu Gui, Bin Gao, Zhipeng Xu, Binbin Zhu, Zhiren Sheng

**Affiliations:** ^1^Department of Anesthesiology, The Affiliated Hospital of Medical School of Ningbo University, Ningbo, China; ^2^Department of Anesthesiology, Affilated Hospital of Shaoxing University, Shaoxing, China; ^3^Department of Radiology, The Affiliated Hospital of Medical School of Ningbo University, Ningbo, China; ^4^Department of Nursing, The Affiliated Hospital of Medical School of Ningbo University, Ningbo, China

**Keywords:** post-pandemic, the tertiary hospital reappraisal, resident doctor, mental health, psychological problem

## Abstract

**Participants:**

Competent resident doctor were expected to help the patients, advance medical knowledge, and promote public health. The time and effort necessary for residents to devote to standarized training is extensive. Anxiety and depression can negatively affect professional development and work efficacy. The study aimed to assess the psychosocial effects of the hospital reappraisal during the post-pandemic era of COVID-19 and analyze potential risk factors leading to their symptoms of anxiety and depression.

**Method:**

In March 2021, the “Questionnaire Star” electronic questionnaire system was used to collect data. A total of 96 resident doctors from the affiliated hospital of the medical school of Ningbo University were invited to complete the questionnaires.

**Results:**

According to our study, the prevalence of symptoms of anxiety and depression in the resident doctors in the institution was 61.5 and 59.4%, respectively. The residents who were worried about clinical skills tend to have anxiety symptoms under online education (OR = 3.436, 95%CI: 1.122–10.526). Compared with participants who were assigned by other hospitals, social trainees (OR: 7.579, 95%CI: 1.747–32.885), and full-time masters (OR: 5.448, 95% CI: 1.586–18.722) were more likely to have anxiety symptoms. Participants without a labor contract (OR = 3.257, 95% CI: 1.052–10.101) had a high risk of depression symptoms. Participants who spent more time learning the details prepared for the tertiary hospital reappraisal were significantly more likely to develop anxiety and depressive symptoms.

**Conclusion:**

This study suggested that the tertiary hospital reappraisal program has an impact on the high incidence of anxiety and depression of the young resident doctors during the post-pandemic era of the COVID-19 in Ningbo.

## Highlights

- As far as we know, few researches aimed to study the effect of a hospital reappraisal program on stress and mental conditions. Our study focused on the issue during the post-pandemic era of the COVID-19 crisis, and we investigated the psychosocial problems of the young residents in Ningbo.
- The results of our study suggested that most of the resident doctors have high degrees of mental problems in our institution, which indicated more effective interventions and support are needed.
- The main risk factors of the residents' mental problems involved both the ramifications of the epidemic, and the impact of the tertiary hospital reappraisal.
- In addition, due to the effective control of the COVID-19, the psychological impact of the regional fluctuation of the pandemic on the trainees was not severe. Besides, Ningbo had been categorized as a low-risk area, so preventive measures such as travel restrictions or making fewer trips outside doesn't have a major impact on the psychological burden of the residents.

## Introduction

Resident doctors are an integral part of clinical teams and are vital to patient care in various clinical settings. Residents progress annually with the advancement of their roles within patient-care teams and participation in increasingly complicated operative cases. These training years are characterized by long work hours and little time for family (1). Meanwhile, clinical residency training primarily emphasizes the development of medical knowledge and technical skills. Yet, non-technical skills (NTS) are also vital to successful clinical practice (2). The NTS include social skills, cognitive skills and personal resource skills, which also contains Managing stress and coping with fatigue (3). Consequently, NTS also contributes to resident doctors have an excellent and efficient performance in work. However, the high incidence of psychological problems is detrimental to doctors' performance and destroys their careers. The medical residency is recognized as a risk period for the development of psychological problems, such as anxiety and depression (4). Resident doctors report the highest rate of having a formally diagnosed mental health condition. This may be because they are in the vulnerable age group when psychiatric disorders start (5). Mounting evidence from many studies suggests that anxiety and depression may affect the daily work of residents and interfere with their non-technical abilities, especially in some stressful situations (5, 6). Studies have consistently shown high levels of anxiety amongst resident physicians (7). To deal with this situation, more measures have been made on a large scale to improve doctors' mental health and fitness (8).

During the COVID-19 pandemic, to quickly control the epidemic and save the lives of infected patients, Chinese doctors have been extremely busy working hard over the past 1 year and great efforts have been made to cope with the tremendous public health crisis (9). In terms of the dangerous epidemic situation, the young resident doctors have experienced varieties of mental health challenges, such as overwork, frustration, loneliness, and other stressors (10). Even the family members of medical staff tend to appear symptoms of anxiety and depression (11). China has responded to COVID-19 in time and efficiently, but the current evidence and published literature on previous epidemics suggest that mental health issues may arise in the post-pandemic era (12). The so-called post-epidemic era does not mean that the epidemic completely disappears and everything goes back to normal as we imagined before. Rather, it means that the epidemic rises and falls, can erupt in small scale at any time, and has a seasonal outbreak (13). Existing evidence indicates that a number of medical health care workers developed mood disorders, anxiety disorders, or posttraumatic stress disorder (PTSD) in the wake of the SARS outbreak in 2003 (14). Therefore, there is an immediate need to identify the long- term mental health consequences of the COVID-19 in the post-pandemic era.

The tertiary hospital reappraisal is a kind of healthcare assessment mechanism of the Chinese government for hospitals. The reappraisal is similar to hospital accreditation, which has been adopted internationally as a way and solution for healthcare quality improvement in hospitals (15). In China, tertiary hospital reappraisal means providing patients with better medical conditions, medical technology and medical services, therefore it also represents the strict evaluation conditions of a tertiary hospital (16). During the period of the reappraisal, the expert groups reviewed the relevant documents and regimens of the hospital, and conduct necessary assessments on the hospital staff. It was a huge challenge for our hospital, which requires the efforts of every doctor, including young residents. The main purpose of the appraisal is to check the quality of health care in different regions, tremendous materials are prepared by young staff for the assessment, and knowledge of the hospital's ability in management, clinical, teaching, and scientific research should be memorized comprehensively (17). This reappraisal extended the working hours of residents to a certain extent

In a word, the mental health of resident doctors should be protected with timely interventions and proper information feedback (18). So far, little attention has been paid to this issue. We conducted a cross-sectional survey to evaluate the psychological conditions of tertiary hospital resident doctors. This work aims to study the effect of a hospital reappraisal program on symptoms of anxiety and depression during the post-pandemic era of the COVID-19 crisis in Ningbo.

## Research Methods

### Research Participants and Study Design

During the period from March 15, 2021 to March 19, 2021, we distributed online questionnaires to 96 resident doctors of the Affiliated Hospital of the medical school of Ningbo University. Subsequently, we received 96 responses accordingly with an effective recovery rate of 100%. At the beginning of the questionnaire, we informed participants that they would be signing the consent by default if they accomplished the survey. All of the residents were invited to voluntarily participate in the online survey. Ethics approval was obtained from the Clinical Ethics Committee of the Affiliated Hospital of the medical school of Ningbo University, and the ethical serial number is KY20210318.

### Study Methods

#### Survey Methods

To prevent the spread of COVID-19 through droplets or contact, we used an online-based survey program “Questionnaire Star” to collect data. The “Questionnaire Star” is an application dedicated to send electronic questionnaires. Researchers can design different options for each question for participants to choose, and they can use web page to answer (10). We explained the purpose, content, and detailed methods of the survey to participants before filling. The content of the questionnaire included general information, problems related to the standardized training, the impact of the tertiary hospital reappraisal and COVID-19 on resident doctors, the mental health of them and so on. All of the questionnaires are anonymous.

#### Measures of Dependent Variables

##### Anxiety Symptoms

We employed the Chinese version of GAD-7 to assess the anxiety symptoms of resident doctors. GAD-7 is a self-report questionnaire that screens and measures the severity of generalized anxiety disorder (19). Participants rated seven items according to the frequency of symptoms in the past 2 weeks on a 4-point scale from 0 (not at all) to 3(nearly every day). Total scores ranged from 0 to 21, with higher scores indicating greater severity of anxiety symptoms. A score of 0–4 has no anxiety, a score of 5–9 may have mild anxiety, a score of 10–13 may have moderate anxiety, and a score of 14–18 may have moderate to severe anxiety, 19-21 may have severe anxiety (20). The GAD-7 has been widely applied in China and good reliability and validity of GAD-7 have been confirmed (21). The presence of mild anxiety symptoms was defined as a total score of ≥5 points in the GAD-7 in this survey (21).

##### Depressive Symptoms

We employed the Chinese version of PHQ-9 to assess the depressive symptoms of the resident doctors. PHQ-9 is a 9-item self-report measure to assess the severity of depression (22). Participants rated each item in accordance with the frequency of symptoms over the past 2 weeks on a 4-point scale from 0 (not at all) to 3 (nearly every day). Total scores ranged from 0 to 27, with the highest scores indicating greater severity of depressive symptoms. A score of 0–4 has no depression, 5–9 may have mild depression, 10–14 may have severe depression, 15–19 may have moderate to severe depression, 20–27 may have severe depression. The PHQ-9 has been widely used in China and good reliability and validity of the Chinese version of PHQ-9 have been demonstrated (23). The mild depressive symptom was defined as a total score of ≥5 points in the PHQ-9 in this survey.

##### Participants Characteristics

We designed the characteristics of the participants on the questionnaire, including gender, grade, major, education background, marital status, whether they have obtained the medical practitioner qualification certificate, whether have signed a contract with a hospital, and so on.

##### The Source of Stress

In the questionnaire, we arranged the options about the source of pressure close to the participants. The questionnaires for the source of stress of participants were self-developed specifically for this study, as there were no suitable scales available for measuring factors related to resident doctors during the post-pandemic era of the COVID-19 crisis. Due to the author's identity as a resident doctor, the following situations were set up for other participants to choose from: (1) numerous examinations; (2) acquiring the knowledge required for the tertiary hospital reappraisal; (3) whether having signed a contract with a hospital; (4) income; (5) Project; (6) COVID-19-related events;(7) interpersonal relationship;(8) the loss of investment; (9) marriage; (10) others. These options contain common sources of stress, which can be supplemented by others to enrich the need of the survey.

##### Ways to Relieve Stress

In the questionnaire, we arranged the options about the ways to relieve stress close to the participants. The questionnaires for the ways to relieve the stress of participants were self-developed specifically for this study, as there were no suitable scales available for measuring factors related to resident doctors during the post-pandemic era of the COVID-19 crisis. The following situations were set up for other participants to choose from: (1) indulge in food; (2) take a rest; (3) take a walk; (4) work; (5) review lessons; (6) chat; (7) go to shopping; (8) drink; (9) sing; (10) travel; (11) play games; (12) Others. These options contain common ways to cope with stress, which can be supplemented by others to enrich the need of the survey.

### Statistical Analysis

This statistical analysis adopted categorical variable statistics. The categorical variables were expressed as percentages, and then the chi-square test is performed to analyze whether there was statistical significance. The binary logistic regression analysis was used to analyze the data of the chi-square test *p* ≤ 0.05 in the categorical variables. Model discrimination and calibration were evaluated using Hosmer–Lemeshow goodness-of-fit statistic. Two-sided *P* < 0.05 was considered statistically significant. These statistical tools included SPSS v25.0 (IBM) and “questionnaire star” to collect statistical data.

## Result

### Demographic Characteristics

Table 1 presents the characteristics of participants. A total of 96 resident doctors from the affiliated hospital of medical school of Ningbo University completed the questionnaire, of whom 51 (53.13%) were men and 45 (46.88%) were women. Among them, 39 resident doctors (40.63%) were from first grade, 23 doctors (23.96%) from second grade, and 34 doctors (35.42%) from third grade. The education level of respondents varied from junior college to master. The participants included a junior college student (1; 1.04%), undergraduate students (61; 63.54%), graduate students (34; 35.42%). Among the participants, 77 (80.21%) were unmarried and 19 (19.79%) were married.

**Table 1:** Sample characteristics and univariate analysis of variables related to symptoms of anxiety and depression.

**Variables**	***n* (%)**	**Anxiety symptoms (GAD-7 score)**		***P***	**Depressive symptoms(PHQ-9 score)**		***P***
		**<5 (*n* = 37)**	**≥5(*n* = 59)**		**<5 (*n* = 39)**	**≥5 (*n*= 5 7)**	
Demographics Gender				**0.572**			**0.594**
Male	51 (53.1)	21 (56.8)	30 (50.8)		22 (56.4)	29 (50.9)	
Female	45 (46.9)	16 (43.2)	29 (49.2)		17 (43.6)	28 (49.1)	
Grade				0.838			0.737
First grade	39 (40.6)	14 (37.8)	25 (42.4)		14 (35.9)	25 (43.9)	
Second grade	23 (24.0)	10 (27.0)	13 (22.0)		10 (25.6)	13 (22.8)	
Third grade	34 (35.4)	13 (35.2)	21 (35.6)		15 (38.5)	19 (33.3)	
Educational background				0.627			0.649
Junior college	1 (1.0)	0 (0.0)	1 (1.7)		0 (0.0)	1 (1.8)	
Undergraduate	61 (63.5)	25 (67.6)	36 (61.0)		26 (66.7)	35 (61.4)	
Master	34 (35.4)	12 (32.4)	22 (37.3)		13 (33.3)	21 (36.8)	
Marital status				0.722			0.504
Spinsterhood	77 (80.2)	29 (78.4)	48 (81.4)		30 (77.0)	47 (82.5)	
Married	19 (19.8)	8 (21.6)	11 (18.6)		9 (23.0)	10 (17.5)	
Fertility circumstance				0.311			0.066
Yes	5 (5.2)	3 (8.1)	2 (3.4)		4 (10.3)	1 (1.8)	
No	91 (94.8)	34 (91.9)	57 (96.6)		35 (89.7)	56 (98.2)	
The type of the standardized training				0.039			0.087
Full-time master	36 (37.5)	8 (21.6)	28 (47.4)		10 (25.6)	26 (45.6)	
Social being	18 (18.8)	9 (24.3)	9 (15.2)		7 (17.9)	11 (19.2)	
Resident doctors assigned by other hospitals	42 (43.8)	20 (54.1)	22 (37.2)		22 (56.5)	20 (35.2)	
Whether have signed a contract with a hospital				0.014			0.004
Yes	60 (62.5)	28 (75.6)	32 (54.2)		26 (66.7)	30 (52.7)	
No	36 (37.5)	9 (24.4)	27 (45.8)		13 (33.3)	27 (47.3)	
Whether the weekly nucleic acid test occupied their leisure time							
				0.035			0.047
Yes	60 (62.5)	28 (75.6)	32 (54.2)		9 (25.6)	31 (54.4)	
No	36 (37.5)	9 (24.4)	27 (45.8)		29 (74.4)	26 (45.6)	
Whether had concerns about clinical skills under the online education							
				0.013			0.401
Yes	37 (38.5)	20 (54.0)	17 (28.8)		17 (43.5)	20 (35.0)	
No	59 (61.5)	17 (46.0)	42 (71.2)		22 (56.5)	37 (65.0)	
The time of acquiring knowledge required for the tertiary hospital reappraisal							
				0.005			0.003
<1 h	18 (18.6)	13 (35.1)	5 (8.5)		14 (35.8)	4 (7.0)	
1–2 h	27 (28.1)	11 (29.8)	16 (27.1)		11 (28.2)	16 (28.0)	
2–3 h	22 (22.9)	7 (18.9)	15 (25.5)		6 (15.5)	16 (28.0)	
>3 h	29 (30.2)	6 (16.2)	23 (38.9)		8 (20.5)	21 (37.0)	

*The bold values indicate the P values for gender*.

### Related Issues During Standardized Training for Residents

In terms of employment, 47 residents have signed contracts with the different hospitals, and the rest of the 49 residents are without labor contracts. Among the participants, 60 residents (62.5%) have obtained the medical practitioner qualification certificate, and the remaining 36 residents (37.5%) have not yet obtained it. The survey also showed that 60 residents (62.5%) took up their rest time due to weekly nucleic acid testing, while 36 (37.5%) did not change their work schedule. After the change of teaching mode due to the epidemic, 37 residents (38.5%) were concerned about the practical skills assessment, and the rest of 59 residents (61.5%) were not concerned. Regarding the time of acquiring knowledge required for the tertiary hospital reappraisal, 18 residents (18.6%) studied for <1 h, 27 residents (28.1%) studied for 1–2 h, 22 residents (22.9%) studied for 2–3 h, and 29 residents (30.2%) studied for more than 3 h.

### Mental Health Status

#### Anxiety Symptoms

The questionnaire suggested that 59 (61.5%) resident doctors in this survey had anxiety-related symptoms. In the logistic regression analysis, several factors were independently associated with anxiety symptoms, such as whether they have obtained the medical practitioner qualification certificate, whether have signed a contract with a hospital, the type of the standardized training, weekly nucleic acid test, whether there is concern about the skill assessment under the online education, and the learning time of acquiring knowledge required for the tertiary hospital reappraisal. However, there was no obvious correlation in gender, educational background, marital status, and grade (Table 2).

**Table 2:** Multivariate logistic regression analysis of variables related to anxiety symptoms.

**Variables**	**Depressive symptoms (PHQ-9 score ≥ 5)**		
	***P***	**OR**	**95%CI**
Whether have signed a contract with a hospital	0.118	2.590	0.786–8.547
Whether have obtained the medical practitioner qualification certificate	0.979	1.017	0.258–3.623
Whether the weekly nucleic acid test occupied their leisure time	0.129	2.294	0.786–6.667
Whether had concerns about clinical skills under the online education	0.031	3.436	1.122–10.526
The time of acquiring knowledge required for the tertiary hospital reappraisal	0.005		
<1 h		Reference	
1–2 h	0.012	6.84	1.536–30.303
2–3 h	0.004	10.86	2.151–55.55
>4 h	0.000	19.231	3.937–90.909
The type of the standardized training	0.004		
Resident doctors assigned by other hospitals		Reference	
Social trainees	0.007	7.579	1.747–32.885
Full-time master	0.007	5.448	1.586–18.722

#### Depressive Symptoms

The questionnaire suggested that 57 (59.4%) resident doctors in this survey had depression-related symptoms. In the logistic regression analysis, several factors were independently associated with depression symptoms, such as whether they have obtained the medical practitioner qualification certificate, whether have signed a contract with a hospital, weekly nucleic acid test, and the learning time of acquiring knowledge required for the tertiary hospital reappraisal. However, there was no obvious correlation in gender, educational background, marital status, grade, the nature of the standardized training, and whether there is concern about the skill assessment under the online education (Table 3).

**Table 3:** Multivariate logistic regression analysis of variables related to depressive symptoms.

**Variables**	**Depressive symptoms (PHQ-9 score ≥ 5)**		
	***P***	**OR**	**95%CI**
Whether have signed a contract with a hospital	0.041	3.257	1.052–10.101
Whether have obtained the medical practitioner qualification certificate	0.403	1.661	0.505–5.464
Whether the weekly nucleic acid test occupied their leisure time	0.248	1.841	0.193–1.529
			0.654–5.181
The time of acquiring knowledge required for the tertiary hospital reappraisa		0.005	
<1 h		Reference	
1–2 h	0.018	5.102	1.319–19.608
2–3 h	0.003	9.346	2.179–40.000
>4 h	0.002	9.174	2.315–37.037

### Multivariate Logistic Regression Analysis of Factors Significantly Associated With Anxiety and Depression Symptoms

#### Anxiety Symptoms

From the above data, we have learned that 61.5% of the resident doctors have symptoms of anxiety and 59.4% of them have symptoms of depression. Multiple logistic regression analysis demonstrated that residents who were worried about clinical skills tend to have anxiety symptoms under online education (OR = 3.436, 95%CI: 1.122–10.526). From the data in Table 2, taking 0–1 h on the study as a reference, compared with 1–2 h (OR = 6.84, 95%CI: 1.536–30.303), 2–3 h (OR = 10.86, 95%CI: 2.151–55.55), and 4 h or more (OR = 19.231, 95%CI: 3.937–90.909), participants who spent more time learning about the acquiring knowledge required for the tertiary hospital reappraisal were significantly more likely to develop anxiety symptoms. Taking participants who assigned by other hospitals as a reference, social being (OR: 7.579, 95%CI: 1.747–32.885) and full-time masters (OR: 5.448, 95% CI: 1.586–18.722) were more likely to have anxiety symptoms.

#### Depressive Symptoms

From the data in Table 3, participants without a contract (OR = 3.257, 95% CI: 1.052–10.101) were significantly more likely to have depression symptoms. Regarding the learning of the acquiring knowledge required for the tertiary hospital reappraisal, taking 0–1 h on the study as a reference, compared with 1–2 h (OR = 5.102, 95%CI: 1.319–19.608), 2–3 h (OR = 9.346, 95%CI: 2.179–40.000), and 4 h or more (OR = 9.174, 95%CI: 2.315–37.037), participants who spent more time on learning were significantly more likely to develop depression symptoms.

## Discussion

As far as we know, resident doctors are a significant part of medical teams and undertake a mass of tedious work (24, 25). Anxiety and depression at work will not only affect their daily life, but also reduce work efficiency and even cause medical accidents (26). This cross-sectional psychological survey suggested that the tertiary hospital reappraisal program has an impact on the high incidence of anxiety and depression of the young resident doctors during the post-pandemic era of the COVID-19 in Ningbo. This study also obtained the factors affecting the psychological condition of the resident doctors in our hospital through a questionnaire and provided suggestions for mitigating the psychological consequences. According to our survey, the prevalence of symptoms of anxiety and depression in the resident doctors in our institution was 61.5 and 59.4%, respectively, which are much higher than the level of the general population in China (27). After controlling for confounders, the main factors affecting residents' mental health are as follows: the worried about clinical skills under the online education; the type of standardized training; whether has signed a labor contract with a hospital; the time of acquiring the knowledge required for the tertiary hospital reappraisal; various examinations; tedious work; low income and so on.

While previous studies mainly focus on the effect of COVID-19 on resident doctors (28, 29), according to the current situation, the regional fluctuation of the pandemic had less impact on the mental health of residents, which was beyond our expectations. The bigger impacts came from the ramifications of the COVID-19 crisis. There were several reasons for this phenomenon. At present, the pandemic in China has been well-controlled through unremitting efforts. The Chinese people have great confidence in the COVID-19 vaccine, and the coverage rate has observably increased (30). Medical supplies such as masks and protective suits are sufficient. Nevertheless, the lack of personal protective equipment (PPE) increased the anxiety of health workers in some countries (31). Furthermore, Chinese authorities adopted early stage integrated psychological crisis interventions following novel corona virus outbreak (32). Besides, the hospital has provided training on COVID-19 prevention for residents, and the impact of COVID-19 on their mental health is gradually diminishing. However, the prevalence of the COVID-19 has changed the way of education (33, 34), and online classes have become the main teaching method for resident doctors (35). In order to prevent the spread of the epidemic, our hospital had also chosen online education as the main teaching strategy to strengthen the training of residents. Doctors need theoretical knowledge as the basis, and they also need to have clinical practice capabilities. Online education may be more focused on the learning of theoretical knowledge, but the young resident doctors require communication and interaction with patients (36). The resources of online education are relatively limited. Compared with teaching in the hospital, online education can provide typical cases and operation specifications (37). However, online learning is helpless in practical training (38). The lack of rehearsal for future operational assessment increased the psychological problems of residents. The unexpected COVID-19 crisis has disorganized medical education, but this may be a seminal opportunity for medical education to develop in the long view (39). Following the COVID-19 pandemic, the revolution of medical education has accelerated. The medical career will put more emphasis on telemedicine, virtual education, and greater national and international cooperation in the future (40). Doctors should be prepared for these changes.

During the period of the tertiary hospital reappraisal, the trainees' spare time was occupied by different levels of transactional work: preparing materials of the daily quality control, arranging documents and photocopies of teaching activities, reciting the inspection-related information and taking part in the reappraisal simulation. This accreditation is beneficial to achieving universal quality health coverage (41), so the criteria of the assessment were very strict, which suggested the complexity of the accreditation (42). As a result of the reappraisal to the hospital staff necessary assessment, repeated exams with various contents increased the pressure on the residents. In addition, most of the residents were assigned by other hospitals, and they were requested to study the acquiring knowledge required for the tertiary hospital reappraisal just to cope with the accreditation. According to our study, the length of study time was positively correlated with the severity of anxiety and depression. In other words, residents who spent more time learning about the acquiring knowledge required for the tertiary hospital reappraisal were significantly more likely to develop the symptoms of anxiety and depression. Due to the need to prepare materials and documents of reappraisal, the working hours of the resident doctors were prolonged. Indeed, several studies have reported that occupational stress, such as excessive workload or working time, was closely related to anxiety and depression (43, 44). To solve the difficulty and accomplish the tasks of the tertiary hospital reappraisal, the hospital manager could encourage the residents to actively participate in the training and give appropriate rewards to the outstanding trainees to strengthen their enthusiasm (45).

Employment is the foundation of the people, and it will generate greater pressure and affect health without work. A large amount of evidence supported that young people are especially vulnerable to mental health problems when unemployed (46, 47). There is essentially no big difference between the type of training and whether have signed a contract with hospitals. They are both employment issues. After the three-years training, the trainees will face the pressure of finding a job competing with fresh graduates, which also caused their anxiety and depression. In addition, lower wages and high-intensity work aggravate the life and work pressure of residents (48). To alleviate the pressure of the trainees, the administrator could increase the rest time of the resident doctors by reasonably planning the work schedule of the trainees, so that the residents have more spare time to regulate their moods. Moreover, the income of trainees can be appropriately increased as overtime subsidies. The pressure of residents both comes from heavy work and frequent tests. In order to cultivate outstanding resident doctors and improve the quality of medical care in China, various assessments of trainees cannot be avoided. The hospital could start several interventions with the aim to optimize the learning skills of trainees and exam preparation to prevent test anxiety, comprising lectures on mental health and study guidance (49). Therefore, the hospital administrators and health authorities could provide efficient interventions with addressing their psychological needs and formulate effective strategies to ameliorate resident doctors' mental health status (50). With the improvement of anxiety and depression problems of the residents, they can work with a more positive attitude to serve patients, which is also conducive to the development of Chinese medical treatment.

## Limitations

The study has limitations. First of all, our research is a single-center study. We collected data based on the resident doctor of the affiliated hospital of the medical school of Ningbo University. The sample size is relatively small, and whether the results are applicable to other tertiary hospitals remains to be further studied. Nevertheless, if the study is clinically significant, it will be used to develop a multicenter project to demonstrate external validity. Secondly, this was a cross-sectional study designed after the outbreak of COVID-19, we're not able to confirm that whether the mental health of resident doctors was more serious by the pandemic with a direct comparison to pre-pandemic conditions. Also, our research was conducted using an anonymous online questionnaire due to the limited research conditions caused by the pandemic, which may have information bias. Finally, the study may be subject to selection bias and the results need to be interpreted with caution.

## Conclusion

According to this cross-sectional survey, most of the resident doctors in our hospital had symptoms of anxiety and depression to varying degrees. The sources of anxiety and depression were similar. Despite of the fact that the regional fluctuation of the pandemic had minorless impact on the mental health of residents, the main sources of psychological burden for residents come from the reduced clinical skills training on account of the impact of COVID-19. Due to the lack of actual practical processes, resident doctors are worried about their practical abilities, yet time after working was spent on the preparation for the tertiary hospital reappraisal, which could accelerate mental problems. The purpose of this survey was to help residents to identify their mental status and think about what need to be done to address their problems prior to any potential mental health conditions developing. More detailed work is urgently needed to explore effective interventions, as well as how we can better understand the needs of resident doctors.

## Data Availability Statement

The original contributions presented in the study are included in the article/supplementary material, further inquiries can be directed to the corresponding author/s.

## Author Contributions

ZSha and YZ performed the experiment. AC, WL, and BZ contributed significantly to analysis and manuscript preparation. ZShe performed the data analyses and wrote the manuscript. JW, YG, BG, ZX, ZShe, and BZ helped perform the analysis with constructive discussions. ZSha and AC contributed equally to this work. All authors contributed to the article and approved the submitted version.

## Funding

This study was funded by joint fund of Affiliated Hospitals and Medical School of Ningbo University (201804), Ningbo Public Welfare Science and Technology Plan Project (202002N3141), Zhejiang Science and Technology Plan of Traditional Chinese Medicine (2020ZB236), Ningbo Science and Technology Innovation 2025 Major Special Project (2019B10035), Core Curriculum Construction Project of Ningbo University School of Medicine.

## Conflict of Interest

The authors declare that the research was conducted in the absence of any commercial or financial relationships that could be construed as a potential conflict ofinterest.

## Publisher's Note

All claims expressed in this article are solely those of the authors and do not necessarily represent those of their affiliated organizations, or those of the publisher, the editors and the reviewers. Any product that may be evaluated in this article, or claim that may be made by its manufacturer, is not guaranteed or endorsed by the publisher.
